# Contrast‐enhanced ultrasound enables precision diagnosis of preoperative muscle invasion in bladder cancer: a prospective study

**DOI:** 10.1002/mco2.70106

**Published:** 2025-02-17

**Authors:** Qiyun Ou, Weibin Xie, Yunfang Yu, Bing Ou, Man Luo, Yongjian Chen, Weiwei Pan, Yiming Lai, Zhuohang Li, Jianqiu Kong, Zhuo Wu, Jingliang Ruan, Jingjing Han, Tianxin Lin, Baoming Luo

**Affiliations:** ^1^ Guangdong Provincial Key Laboratory of Malignant Tumor Epigenetics and Gene Regulation, Department of Ultrasound in Medicine, Department of Urology, Department of Medical Oncology Sun Yat‐sen Memorial Hospital Sun Yat‐sen University Guangzhou Guangdong China; ^2^ Department of Oncology Nanfang Hospital Southern Medical University Guangzhou Guangdong China; ^3^ Guangdong Provincial Clinical Research Center for Urological Diseases Guangzhou Guangdong China; ^4^ Shenshan Medical Center Sun Yat‐sen Memorial Hospital Sun Yat‐sen University Shanwei Guangdong China; ^5^ Faculty of Medicine Macau University of Science and Technology Taipa Macao China; ^6^ Department of Medical Oncology The Third Affiliated Hospital of Sun Yat‐sen University Guangzhou Guangdong China

**Keywords:** bladder cancer, contrast‐enhanced ultrasound, muscle invasion, precision diagnosis, prospective study

## Abstract

Bladder cancer's high mortality underscores the need for precise staging, especially to differentiate between nonmuscle invasive bladder cancer (NMIBC) and muscle invasive bladder cancer (MIBC) types. This prospective study evaluated the efficacy of contrast‐enhanced ultrasound (CEUS) for preoperative staging, focusing on its ability to distinguish NMIBC from MIBC. Conducted from April 2020 to September 2021, the study involved 163 patients (median age: 64.0 years; 137 males, 26 females), with 133 NMIBC (81.6%) and 30 MIBC (18.4%). Each patient underwent CEUS followed by transurethral resection of bladder tumor or radical cystectomy. CEUS demonstrated high diagnostic accuracy in determining muscle invasion status (sensitivity 83.3%, specificity 92.5%, accuracy 90.8%, area under the receiver operating characteristic curve [AUC] 0.88). Comparative analyses against MRI (AUC 0.77) showed CEUS outperforming in muscle invasion detection. Combining CEUS with MRI improved diagnostic accuracy, particularly when MRI vesical imaging reporting and data system score was 3 points. The combined approach achieved an AUC of 0.73, with sensitivity, specificity, and accuracy of 76.2, 70.2, and 71.6%, respectively. Thus, CEUS emerges as a valuable diagnostic tool for preoperative staging of bladder cancer, particularly in its role in assessing muscle invasion status and thereby aiding in clinical decision‐making and intervention outcomes.

## Graphical abstract

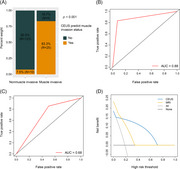

## 1 INTRODUCTION

Bladder cancer is the 10^th^ most common cancer worldwide, with approximately 573,000 new detected cases and 213,000 deaths recorded in 2020.^1^ Bladder cancer staging is critical in determining therapeutic strategy. Bladder cancer can be divided into nonmuscle invasive bladder cancer (NMIBC) and muscle invasive bladder cancer (MIBC), with the latter characterized by high aggressiveness, complicated treatment, high propensity to metastasis, and poor prognosis.^2^ In clinical practice, radical cystectomy is performed on MIBC patients, and transurethral resection of bladder tumor (TURBT) is performed on NMIBC patients. Patients suspected to have remaining MIBC after TURBT surgery require secondary TURBT.^2^

Cystoscopy biopsy determines the pathological classification of the lesions, but it has limitations in diagnosing muscular infiltration, especially in the case of large lesions. The current recommendation is that patients with high‐grade bladder tumors (more aggressive tumors) or those with abnormal urine cytology findings should undergo random biopsies.^3^ This additional diagnostic step assists healthcare providers in acquiring additional information about the tumor, which is crucial for devising the most suitable treatment strategy.

The magnetic resonance imaging (MRI)‐based standardized vesical imaging reporting and data system (VI‐RADS) protocol is generally acknowledged as the gold standard for predicting muscle invasion in bladder cancer.^4^ However, its use has been limited by procedure‐related contraindications and the fact that it should be avoided in patients with renal damage, especially in cases of renal failure.^4^

Ultrasound can be used to detect and stage bladder cancers.^5^, ^6^ Contrast‐enhanced ultrasound (CEUS) identifies the vascularity of tissues and organs. It can provide more abundant and accurate diagnostic information than conventional ultrasound and color Doppler ultrasound. Moreover, it is characterized by the absence of radiation exposure or previously known physically harmful interactions with tissues, lack of risks in case of renal insufficiency, lower incidence of adverse reactions, wide accessibility, noninvasiveness, and affordability compared with MRI.^7^, ^8^, ^9^ Continuous innovation of CEUS technology, parametric micro‐flow imaging (P‐MFI), MFI, superb microvascular imaging, time curve analysis, and other related auxiliary functions have allowed to acquire more information on diseases while greatly improving the diagnostic accuracy of this technique. However, the accuracy of preoperative muscle invasion detection in bladder cancer using CEUS remains unclear. The present study investigated the value of CEUS in preoperative detection and differentiation between NMIBC compared with MIBC in diagnosed patients.

## 2 RESULTS

### 2.1 Patient characteristics

**FIGURE 1:**
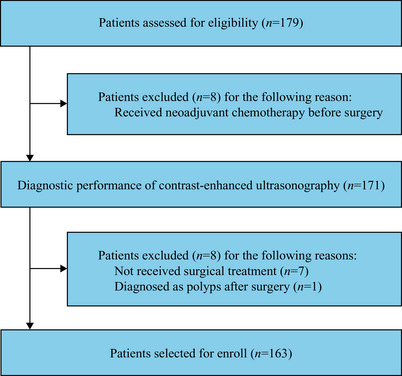
Study flow diagram.

A total of 163 patients were evaluated for study eligibility (Figure 1). The cohort had a median age of 64.0 years (interquartile range [IQR] 56.0–72.0), and consisted of 137 males (84.0%) and 26 females (16.0%) patients with pathologically confirmed bladder cancer. Among these patients, 133 (81.6%) were diagnosed with NMIBC, while 30 (18.4%) had MIBC and were prospectively enrolled. In addition, 130 (79.8%) patients had a high pathology grade, while 33 (20.2%) had a low pathology grade. Further details regarding the patients’ clinicopathological characteristics are provided in Table 1.

**TABLE 1:** Clinicopathologic characteristics of all patients.

Characteristic	*N* = 163
Gender (%)	
Male	137 (84.0)
Female	26 (16.0)
Age, years, median (IQR)	64.0 (56.0, 72.0)
Gross hematuria (%)	
No	35 (21.5)
Yes	128 (78.5)
Number of urine red blood cell (median (IQR)	139 (11, 1353)
Tumor size, median (IQR)	30 (19, 44)
Tumor size (%)	
<30 mm	81 (49.7)
≥30 mm	82 (50.3)
Tumor base structure status (%)	
Pedunculated	131 (80.4)
Sessile	32 (19.6)
Tumor shape (%)	
Papillary or cauliflower‐like	130 (79.8)
Irregularity	33 (20.2)
Number of tumors (%)	
1	97 (59.5)
2	20 (12.3)
≥3	46 (28.2)
Bladder base continuity status (%)	
Good continuity	128 (78.5)
Discontinuous change	35 (21.5)
Tumor base width (%)	
Narrow	122 (74.8)
Broad	41 (25.2)
Tumor blood flow situation (%)	
Less	120 (73.6)
Rich	43 (26.4)
Bladder cancer muscle invasive status	
NMIBC	133 (81.6)
MIBC	30 (18.4)
Bladder cancer pathology grade	
Low	33 (20.2)
High	130 (79.8)

Abbreviations: IQR, interquartile range; MIBC, muscle invasive bladder cancer; NMIBC, nonmuscle invasive bladder cancer.

### 2.2 CEUS characteristics associated with muscle invasion and pathology grade status

**FIGURE 2:**
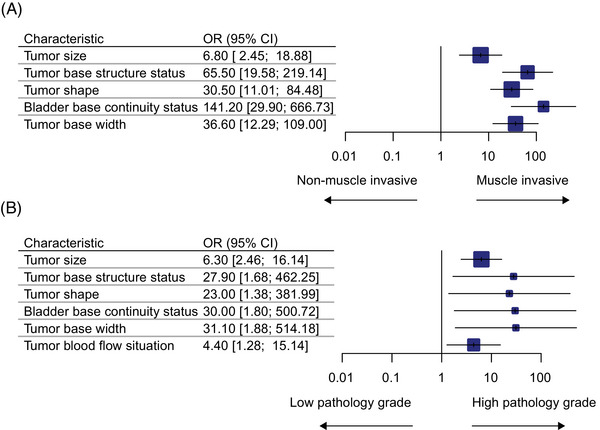
Multivariate analysis of CEUS for the bladder cancer muscle invasion status and pathology grade status. CEUS, contrast‐enhanced ultrasound. Multivariate analysis of CEUS for the bladder cancer muscle invasion status; (B) multivariate analysis of CEUS for the bladder cancer pathology grade status. CEUS, contrast‐enhanced ultrasound.

The univariate analysis of the bladder cancer muscle invasion status in relation to clinicopathological characteristics is shown in Table 2. The median tumor size in MIBC (43 mm [IQR 32–56 mm]) was significantly larger than that in NMIBC (28 mm [IQR 17–40]) (≥30 mm [83.3%] vs. [42.9%], *p* < 0.001), and the area under the receiver operating characteristic curve (AUC) of the individual tests in predicting muscle invasion status were 0.70 (Figure S1). The MIBC patients had significantly higher rates of tumor sessile structures (86.7 vs. 9.0%, *p* < 0.001), more irregular tumor shapes (73.3 vs. 8.3%, *p* < 0.001), more discontinuous changes in bladder base (93.3 vs. 9.0%, *p* < 0.001), and broader tumor bases (83.3 vs. 12.0%, *p* < 0.001) than the NMIBC patients, achieving AUCs of 0.89, 0.83, 0.92, and 0.86 in predicting muscle invasion status, respectively (Figures S2–S5). Multivariable analysis showed that the tumor size, tumor base structure status, tumor shape, bladder base continuity status, and tumor base width CEUS characteristics were independent predictors of the bladder cancer muscle invasion status (all *p* < 0.001; Figure 2A). Moreover, tumor size, tumor base structure status, tumor shape, bladder base continuity status, tumor base width, and tumor blood flow condition were closely associated with the pathology grade status of bladder cancer (all *p* < 0.05; Figure 2B).

**TABLE 2:** Univariate analysis of the bladder cancer muscle invasion status in relation to clinicopathologic characteristics.

	NMIBC group	MIBC group	
Characteristic	*N* = 133	*N* = 30	*p* value
Gender (%)			0.134^b^
Male	115 (86.5)	22 (73.3)	
Female	18 (13.5)	8 (26.7)	
Age, years, median (IQR)	63.0 (56.0, 72.0)	69.5 (61.0, 72.8)	0.109^a^
Gross hematuria (%)			0.977
No	28 (21.1)	7 (23.3)	
Yes	105 (78.9)	23 (76.7)	
Number of urine red blood cell, median (IQR)	60.0 (7.0, 1362.0)	477.5 (192.3, 1196.3)	0.016^a^
Tumor size, median (IQR, cm)	28(17, 40)	43 (32, 56)	<0.001^a^
Tumor size (%)			
<30 mm	76 (57.1)	5 (16.7)	<0.001
≥30 mm	57 (42.9)	25 (83.3)	
Tumor base structure status (%)			<0.001
Pedunculated	121 (91.0)	4 (13.3)	
Sessile	12 (9.0)	26 (86.7)	
Tumor shape (%)			<0.001
Papillary or cauliflower‐like	122 (91.7)	8 (26.7)	
Irregularity	11 (8.3)	22 (73.3)	
Number of tumors (%)			0.164^b^
1	78 (58.6)	19 (63.3)	
2	14 (10.5)	6 (20.0)	
≥3	41 (30.8)	5 (16.7)	
Bladder base continuity status (%)			<0.001
Good continuity	121 (91.0)	2 (6.7)	
Discontinuous change	12 (9.0)	28 (93.3)	
Tumor base width (%)			<0.001
Narrow	117 (88.0)	5 (16.7)	
Broad	16 (12.0)	25 (83.3)	
Tumor blood flow situation (%)			0.100
Less	102 (76.7)	18 (60.0)	
Rich	31 (23.3)	12 (40.0)	

Abbreviations: IQR, interquartile range; MIBC, muscle invasive bladder cancer,

^a^ Wilcoxon rank‐sum,

^b^ fisher's exact test; NMIBC, nonmuscle invasive bladder cancer.

The tumor size of high‐grade bladder cancer (36 mm [IQR 22–47]) was relatively bigger than that of low‐grade bladder cancer (20 mm [IQR 14–27]) (≥30 vs. < 30 mm, *p* < 0.001). Pedunculated lesions accounted for the majority of the low‐grade bladder cancers, among which their proportion was much higher than that among the high‐grade bladder cancers (100.0 vs. 74.6%, *p* = 0.001). The high‐grade bladder cancer patients had significantly higher rates of irregular tumor shape (25.4 vs. 0.0%, *p* = 0.003), more discontinuous changes in bladder base (30.8 vs. 0.0%, *p* = 0.002), a broader tumor base (31.5 vs. 0.0%, *p* < 0.001), and increased tumor blood flow (30.8 vs. 9.1%, *p* = 0.021) than the low‐grade bladder cancer patients. Multivariable analysis showed that CEUS characteristics of tumor size, tumor base structure status, tumor shape, bladder base continuity status, tumor base width, and tumor blood flow condition CEUS characteristics were independent predictors of bladder cancer pathology grade status (all *p* < 0.05). More details on univariate and multivariate analyses are shown in Table 3 and Figure 2B.

**TABLE 3:** Univariate analysis of the bladder cancer pathology grade status in relation to clinicopathologic characteristics.

	LPG group	HPG group	
Characteristic	*N* = 33	*N* = 130	*p* value
Gender (%)			0.510
Male	26 (78.8)	111 (85.4)	
Female	7 (21.2)	19 (14.6)	
Age, years, median (IQR)	63.0 (57.0, 67.0)	64.0 (56.0, 72.0)	0.504^a^
Gross hematuria (%)			1.000
No	7 (21.2)	28 (21.5)	
Yes	26 (78.8)	102 (78.5)	
Number of urine red blood cell, median (IQR)	10.0 (0.0, 87.0)	246.5 (23.0, 1612.0)	<0.001^a^
Tumor size, median (IQR)	20 (14, 27)	36 (22, 47)	<0.001^a^
<30 mm	27 (81.8)	54 (41.5)	
≥30 mm	6 (18.2)	76 (58.5)	
Tumor base structure status (%)			0.001
Pedunculated	33 (100.0)	97 (74.6)	
Sessile	0 (0.0)	33 (25.4)	
Tumor shape (%)			0.003
Papillary or cauliflower‐like	33 (100.0)	97 (74.6)	
Irregularity	0 (0.0)	33 (25.4)	
Number of tumors (%)			0.115^b^
1	24 (72.7)	73 (56.2)	
2	1 (3.0)	19 (14.6)	
≥3	8 (24.2)	38 (29.2)	
Bladder base continuity status (%)			0.002
Good continuity	33 (100.0)	90 (69.2)	
Discontinuous change	0 (0.0)	40 (30.8)	
Tumor base width (%)			<0.001
Narrow	33 (100.0)	89 (68.5)	
Broad	0 (0.0)	41 (31.5)	
Tumor blood flow situation (%)			0.021
Less	30 (90.9)	90 (69.2)	
Rich	3 (9.1)	40 (30.8)	

Abbreviations: HPG, high pathology grade; IQR, interquartile range; LPG, low pathology grade,

^a^ Wilcoxon rank‐sum,

^b^ fisher's exact test.

### 2.3 High overall CEUS diagnostic performance in muscle invasion status evaluation

**FIGURE 3:**
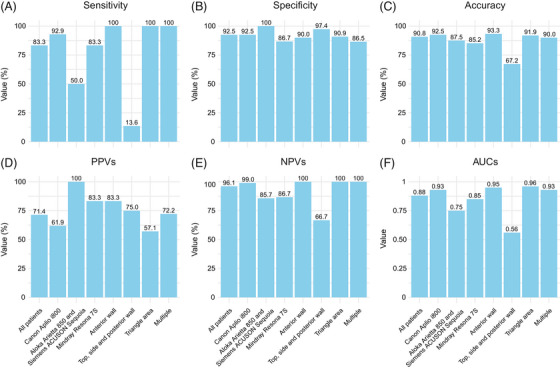
Diagnostic performance of CEUS for the muscle invasion status in bladder cancer. CEUS, contrast‐enhanced ultrasound. CEUS sensitivity for muscle invasion status in bladder cancer with different machines (such as Canon Aplio i800, Aloka Arietta 850 and Siemens ACUSON Sequoia, Mindray Resona 7S) and lesion locations (such as anterior wall, top, side and posterior wall, triangle area, multiple lesions); (B) CEUS specificity for muscle invasion status in bladder cancer with different machines (such as Canon Aplio i800, Aloka Arietta 850 and Siemens ACUSON Sequoia, Mindray Resona 7S) and lesion locations (such as anterior wall, top, side and posterior wall, triangle area, multiple lesions); (C) CEUS accuracy for muscle invasion status in bladder cancer with different machines (such as Canon Aplio i800, Aloka Arietta 850 and Siemens ACUSON Sequoia, Mindray Resona 7S) and lesion locations (such as anterior wall, top, side and posterior wall, triangle area, multiple lesions); (D) CEUS PPVs for muscle invasion status in bladder cancer with different machines (such as Canon Aplio i800, Aloka Arietta 850 and Siemens ACUSON Sequoia, Mindray Resona 7S) and lesion locations (such as anterior wall, top, side and posterior wall, triangle area, multiple lesions); (E) CEUS NPVs for muscle invasion status in bladder cancer with different machines (such as Canon Aplio i800, Aloka Arietta 850 and Siemens ACUSON Sequoia, Mindray Resona 7S) and lesion locations (such as anterior wall, top, side and posterior wall, triangle area, multiple lesions); (F) CEUS AUC for muscle invasion status in bladder cancer with different machines (such as Canon Aplio i800, Aloka Arietta 850 and Siemens ACUSON Sequoia, Mindray Resona 7S) and lesion locations (such as anterior wall, top, side and posterior wall, triangle area, multiple lesions). CEUS, contrast‐enhanced ultrasound; PPVs, positive predictive values; NPVs, negative predictive values; AUC, area under the receiver operating characteristics curve.

**FIGURE 4:**
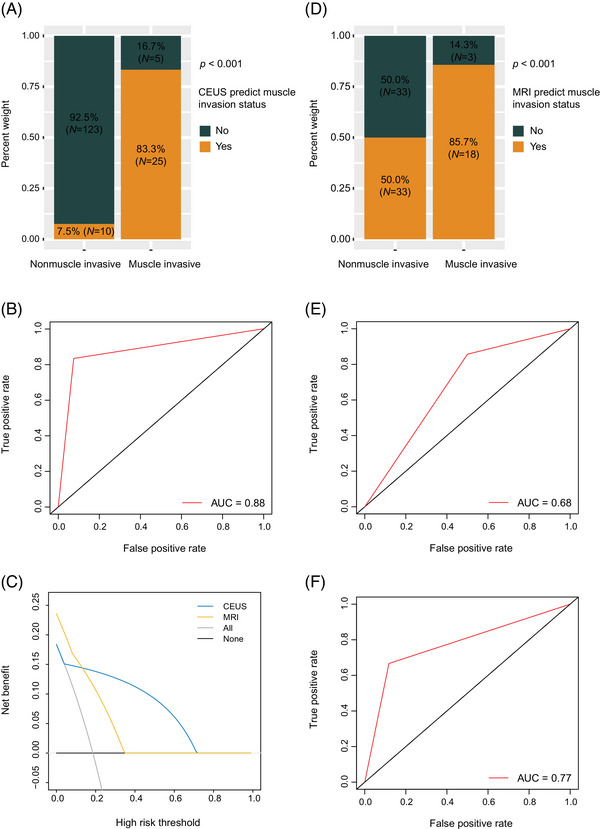
CEUS and MRI for detection of muscle invasion status in bladder cancer. CEUS, contrast‐enhanced ultrasound, MRI, magnetic resonance imaging. (A) Proportions of CEUS in patients with nonmuscle invasive versus muscle invasive bladder cancer; (B) CEUS for bladder cancer muscle invasion status estimation; (C) decision curve analysis; (D) proportions of MRI in patients with nonmuscle invasive versus those with muscle invasive cancer; (E) MRI results for bladder cancer muscle invasion status estimation; (F) CEUS prediction of muscle invasion status for identification of MRI prediction error. CEUS, contrast‐enhanced ultrasound; MRI, magnetic resonance imaging.

The sensitivities, specificities, accuracies, positive predictive values (PPVs), negative predictive values (NPVs), and AUCs of the individual tests in predicting muscle invasion status are summarized in Figure 3A–F. The CEUS demonstrated superior performance in muscle invasion status diagnosis (83.3% [25/30] vs. 7.5% [10/133] and independent samples *t*‐test, *p* < 0.001; Figure 4A), as well as high AUC (0.88, 95% confidence interval (CI) 0.81–0.95; Figure 4B), sensitivity (83.3%), specificity (92.5%), and accuracy (90.8%).

Subgroup analyses of CEUS diagnostic performance in muscle invasion status evaluation are shown in Figure 3A–F. The diagnostic performance of CEUS in determining muscle invasion status in bladder cancer patients is primarily influenced by the following subgroups: different machines may lead to AUC performance heterogeneity, with scores of 0.93 (95% CI 0.85–1.00) for Canon Aplio, 0.75 (95% CI 0.47–1.00) for Aloka Arietta 850 and Siemens ACUSON Sequoia, and 0.85 (95% CI 0.71–0.99) for Mindray Resona 7S. Lesions in the anterior wall (AUC: 0.95, 95% CI 0.85–1.00) and the triangle area (AUC: 0.96, 95% CI 0.91–1.00) were better diagnosed than those located in the top, side, or posterior walls (AUC: 0.56, 95% CI 0.48–0.63), indicating that lesions location may also affect diagnostic accuracy.

The decision curve analysis (DCA) curve showed that the net benefit of using CEUS was significantly higher than that of using MRI if the threshold probability was between 14 and 72%. This suggests that using CEUS for predicting patients’ muscle invasion status was more beneficial than MRI (Figure 4C).

### 2.4 Comparative diagnostic performance of CEUS and MRI in muscle invasion detection in bladder cancer

**FIGURE 5:**
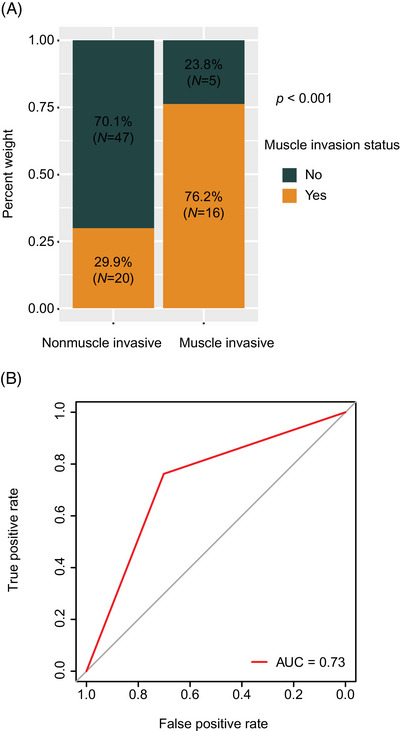
Combining CEUS and MRI to predict muscle invasion status, where CEUS results were used when MRI VI‐RADS score was 3 points. CEUS, contrast‐enhanced ultrasound, MRI, magnetic resonance imaging, VI‐RADS, vesical imaging reporting and data system. The proportions of combining results of CEUS and MRI in patients with nonmuscle invasive versus those with muscle invasive; (B) combining results of CEUS and MRI for bladder cancer muscle invasion status estimation. CEUS, contrast‐enhanced ultrasound; MRI, magnetic resonance imaging.

MRI VI‐RADS has been validated for the detection of muscle invasion in bladder cancer. Bladder cancer lesions classified as VI‐RADS 3 and above by MRI cannot exclude the possibility of muscle infiltration.^10^ Diagnostic performance comparison between CEUS and MRI for the prediction of bladder cancer stage indicated that the MRI performance in preoperative diagnosis of the muscle invasion status was 85.7% [18/21] versus 50.0% [34/68] (*p* = 0.008; Figure 4D) for the detection of the presence or absence of muscular infiltrates. MRI showed an AUC of 0.68 (Figure 4E), and its overall diagnostic sensitivity, specificity, and accuracy were values 85.7, 50.0, and 58.4%, respectively. If MRI results are inconsistent with pathological findings, using CEUS results instead yields a combined diagnostic performance with a higher AUC score of 0.77 (Figure 4F) compared with using MRI alone. Based on our results, if MRI VI‐RADS score of 3 was considered to indicate muscle infiltration, there were 33 cases where the MRI results were inconsistent with the pathology results, while the CEUS outcomes were consistent with the pathology results. Correspondingly, there were only five cases misjudged by CEUS using pathology as a reference, while the MRI results were consistent with the pathology results. An attempt was made to combine CEUS with MRI, such that the CEUS results prevailed when the MRI VI‐RADS score was 3 points. The combined results showed a higher AUC score of 0.73 compared with MRI alone, with an overall diagnostic sensitivity, specificity, and accuracy values of 76.2, 70.1, and 71.6%, respectively (Figure 5).

### 2.5 Case studies: CEUS characteristics and pathological features in bladder cancer patients

**FIGURE 6:**
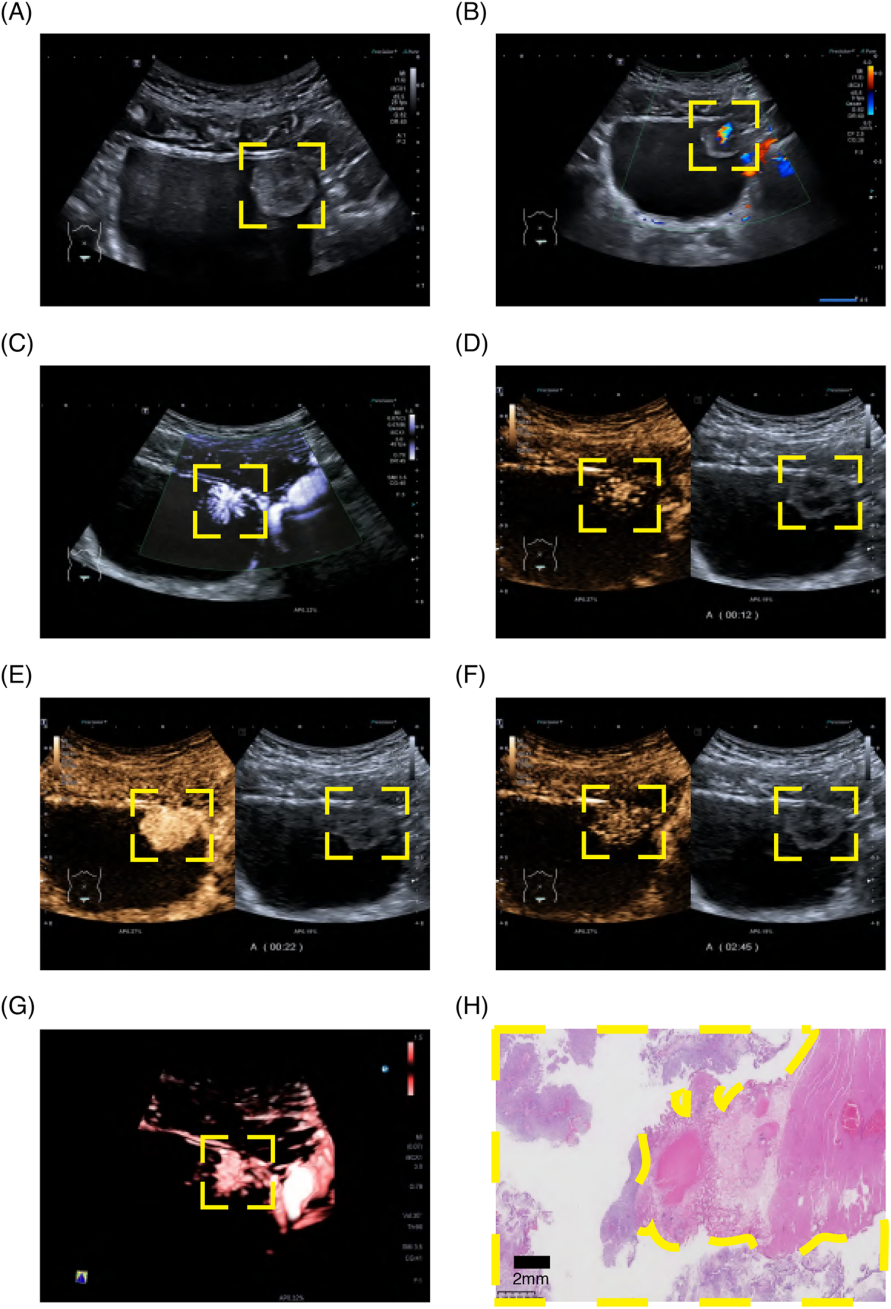
Ultrasound characteristics and pathology status of nonmuscle invasive bladder cancer patients and pathology status. (A) Gray‐scale ultrasound; (B) color Doppler flow imaging; (C) contrast harmonic imaging + superb micro vascular imaging; (D) contrast harmonic imaging in 12 s just before the lesion began to strengthen; (E) contrast harmonic imaging in 22 s almost before the lesion reached the peak; (F) contrast harmonic imaging in 165 s after contrast material was washed out; (G) contrast harmonic imaging + superb micro vascular imaging + smart 3D; (H) pathology imaging. The yellow boxes highlight the lesions.

**FIGURE 7:**
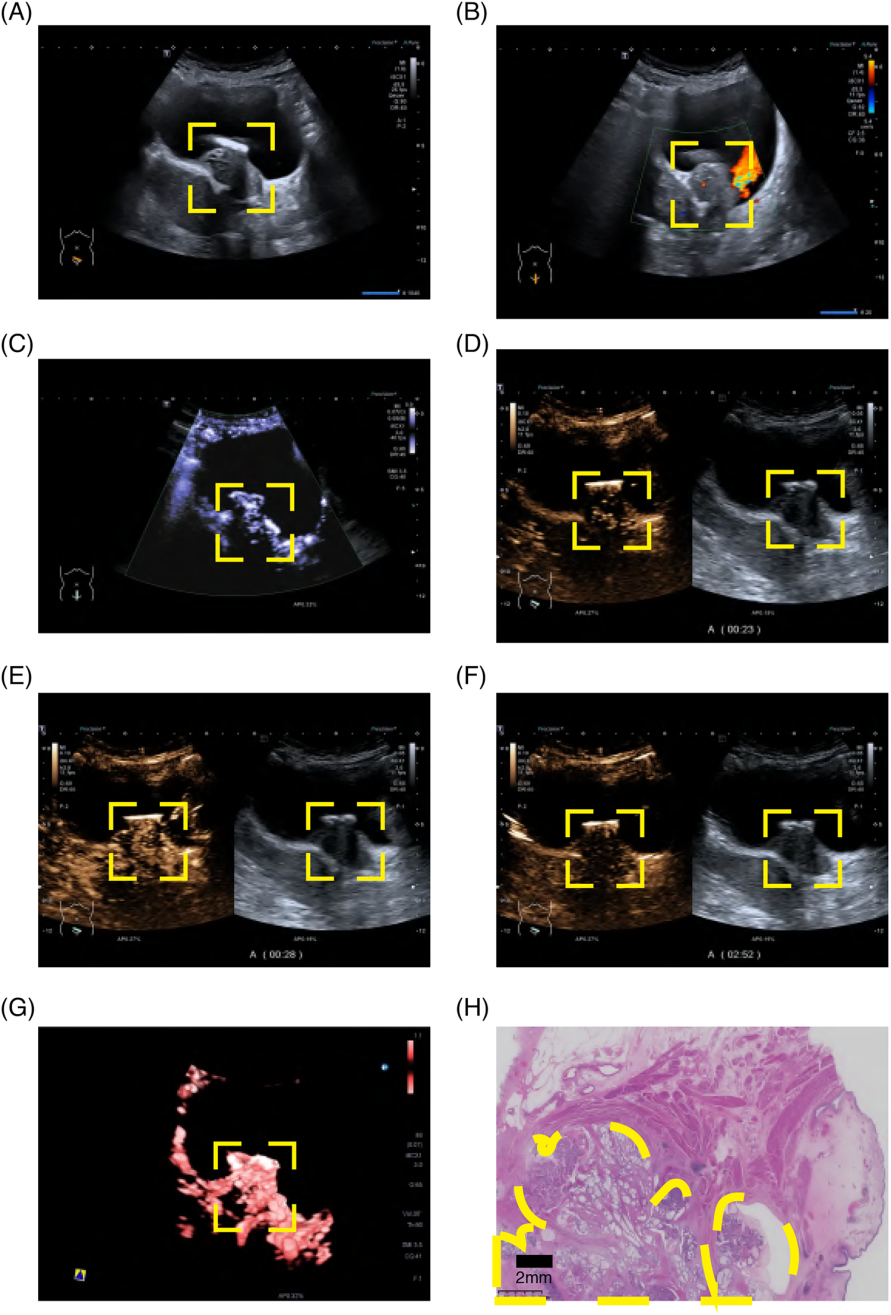
Ultrasound characteristics of muscle invasive bladder cancer patients and pathology status. (A) gray‐scale ultrasound; (B) color doppler flow imaging; (C) contrast harmonic imaging + superb micro vascular imaging; (D) contrast harmonic imaging in 23 s just after the lesion begins to strengthen; (E) contrast harmonic imaging in 28 s almost before the lesion reaches the peak; (F) contrast harmonic imaging in 172 s after contrast material has washed out; (G) contrast harmonic imaging + superb micro vascular imaging + smart 3D; (H) pathology imaging. The yellow boxes highlight the lesions.

Patient 1 was determined to have NMIBC by CEUS. The postoperative pathology confirmed nonmuscle invasive status, gray‐scale ultrasound showed good continuity of muscular layer at the base of the lesion, while CEUS revealed that the muscular layer at the lesion base was intact and without interruption (Figure 6A–H and Video S1). In contrast, patient 2 was diagnosed with MIBC by CEUS. The postoperative pathology confirmed the muscle invasive status, and gray‐scale ultrasound and CEUS showed that the muscular layer at the base was interrupted by the lesion (Figure 7A–H and Video S2). Dynamic observation with CEUS allowed the visualization of the bladder wall divided into three different areas corresponding to the enhanced mucosal layer and muscularis propria, nonenhanced muscular layer, and enhanced serosal layer.

## 3 DISCUSSION

This prospective study showed that bladder imaging using CEUS is highly accurate in preoperative staging. CEUS may achieve a more accurate diagnosis of muscular infiltration compared with MRI.

Neoadjuvant cisplatin‐based combined chemotherapy followed by total cystectomy is the main treatment for MIBC.^2^, ^11^ VI‐RADS is a standardized system for acquiring and interpreting images to evaluate MIBC using multiparametric MRI.^10^ It was developed and jointly released in 2018 by the Japanese Society of Abdominal Radiology, European Association of Urology, and European Society of Urogenital imaging.^10^ VI‐RADS is a structured five‐point risk stratification system that categorizes the likelihood of muscle invasion in bladder cancer patients, ranging from least to most likely: VI‐RADS 1 (highly unlikely), VI‐RADS 2 (unlikely), VI‐RADS 3 (equivocal), VI‐RADS 4 (likely), and VI‐RADS 5 (very likely).^10^ A systematic review, encompassing 20 studies (*n* = 1724), revealed that MRI demonstrated ultimate sensitivity and specificity, achieving 0.92 and 0.88, respectively, in distinguishing between NMIBC and MIBC.^12^ VI‐RADS has attracted significant attention due to its ability to provide a quantitative assessment of the risk of MIBC risk.^13^

Compared with MRI, ultrasound offers several advantages, including real‐time dynamic observation, low incidence of adverse reactions, wide accessibility, noninvasiveness, and cost effectiveness. It has been widely used in various clinical fields, such as abdominal, urinary, reproductive, and hepatobiliary system intervention, the latter including liver or portal vein thrombosis biopsy, liver or kidney ablation, cholangiography, and kidney or prostate biopsy.^14^, ^15^, ^16^ Additionally, ultrasound also plays a significant role in bladder cancer staging, allowing discrimination of different types and stages of bladder cancers.^5^ A systematic review and meta‐analysis of CEUS effectiveness in bladder cancer indicated that it distinguishes T staging and grading of bladder cancer with sensitivity, specificity, and diagnostic AUC of >90.0%.^12^

The present prospective study found that the CEUS bladder imaging is highly accurate in preoperative staging, particularly outperforming MRI in diagnosing muscular infiltration, with an AUC of 0.88, sensitivity of 83.3%, specificity of 92.5%, and overall accuracy of 90.8%. The high level of diagnostic performance of CEUS was validated in many patient subgroups. According to visual observation and radiologist's experience, muscular layer infiltration can be diagnosed when the continuity of the low‐enhancement muscular layer is interrupted. If the continuity of the muscular layer is preserved, the lesion can be judged as not infiltrating the muscular layer.

In addition, the diagnostic performance of CEUS in bladder cancer stage prediction was compared with that of MRI. Combined CEUS‐based diagnosis, where ultrasound results were used to replace MRI results in cases of discrepancies were found between MRI findings and postoperative pathology findings, demonstrated higher AUC (0.77) performance compared with that of MRI alone. This suggests that CEUS can identify muscle invasion statuses that was misdiagnosed by MRI before surgery.

DCA was used to compare the performance of different models and determine the threshold probability at which using the model is most beneficial. The DCA curve showed that the net benefit of using CEUS was significantly higher than that of using MRI if the threshold probability was between 14 and 72%. This demonstrated that the use of CEUS to predict muscle invasion status was more beneficial than MRI‐based diagnosis. Moreover, CEUS can be used for real‐time dynamic observations that can reveal the vascularity of tissues and organs and allow for contrast agent perfusion processes. These features can provide more comprehensive and accurate diagnostic information than conventional ultrasound and color Doppler ultrasound examinations. In addition, CEUS is characterized by lower incidence of adverse reactions, wide accessibility, noninvasiveness, and affordability compared with MRI.^17^, ^18^

It is also noteworthy that the present study results showed that CEUS accuracy varies across different instruments. The Aplio i800 offered exceptional diagnostic performance as it showed the highest AUC. This may be attributed to its auxiliary functions, such as P‐MFI and MFI, which effectively display the enhancement range and timing of lesions. P‐MFI is a post‐contrast processing imaging function that utilizes time to encode the time sequence of contrast agent perfusion. Therefore, P‐MFI can reveal whether a lesion is enhanced earlier than the bladder wall and the overall enhancement lesion stage. This helps to determine whether a lesion is benign or malignant and to identify the lesion. Auxiliary examinations, such as superb microvascular imaging, color superb microvascular imaging, monochromatic superb microvascular imaging, and Smart 3D, help in understanding the overall blood supply of a lesion, which contributes to staging and grading it. It is remarkable that the use of equipment employing different imaging technologies, data transmission systems, image quality or functions, among other characteristics, resulted in different diagnostic accuracies. Specifically, factors such as spatial resolution and frame rate are crucial for diagnostic precision. The discrepancies in image quality are particularly pronounced for lesions located in different bladder locations. Using similar probe frequencies, the distance between lesions and skin surface are considered different, which affects the lateral resolution, resulting in image quality variability, and diagnostic accuracy differences. Specifically, the energy of ultrasonic wave in the transmission process decreases with the increase in the propagation distance, which also decreases the image quality. Thus, choosing the right probe with a proper frequency can help to improve diagnostic accuracy.

Besides, this prospective study's results indicate that lesions in the anterior wall (AUC: 0.95, 95% CI 0.85–1.00) and the triangle area (AUC: 0.96, 95% CI 0.91–1.00) are diagnosed with higher accuracy compared with lesions located in the top, side, or posterior walls (AUC: 0.56, 95% CI 0.48–0.63). This variation in diagnostic accuracy can be attributed to several factors. Lesions in the top region are often affected by lateral wall echo drop‐out and interference from bowel gas, which can obscure the image. Sidewall lesions are primarily impacted by echo drop‐out, leading to decreased visibility. Posterior lesions face challenges due to their greater distance from the probe, resulting in reduced lateral resolution. Conversely, lesions in the anterior wall and triangle area benefit from their closer proximity to the probe and superior lateral resolution, making them more clearly visible and easier to diagnose.

Despite its promising results, the present study had several limitations. First, the diagnosis of sessile structures, irregular tumor shapes, broader tumor bases, and tumor blood flow was based on subjective evaluation, which could introduce variability. Future studies should incorporate quantitative metrics and standardized evaluation criteria to mitigate this bias. Additionally, potential confounding variables such as patient age and prior treatment history were not controlled for in the present study. Future research should account for these variables to isolate the effect of CEUS on diagnostic accuracy.

Second, due to differences in hardware conditions such as imaging technology and spatial resolution, diagnostic accuracy may vary between different devices. Therefore, future research should aim to use the same machine to acquire ultrasound contrast images and make diagnoses for patients in order to control potential confounding variables and thereby increase the reliability of assessing diagnostic accuracy.

Third, transabdominal CEUS's effectiveness in deep position examinations was limited due to decreased lateral resolution at greater depths, particularly for masses on the posterior bladder wall. In addition, the evaluation of the top bladder wall was hindered by lateral wall echo drop‐out and bowel gas interference, while the lateral bladder wall assessment was mainly affected by lateral echo loss. This limitation may explain why the AUC for diagnosing lesions on the top, side, and posterior wall was 0.56, significantly lower than that for lesions on the anterior wall and in the bladder triangle area. To overcome this limitation, combining transabdominal CEUS with transrectal or transvaginal CEUS could be considered in the future to further improve the diagnostic accuracy of bladder cancer staging.

Furthermore, metabolic profiling holds significant scientific value in predicting cancer immunotherapy effectiveness.^19^ Developing a radiomics model can predict the risk of metastasis in cancer patients.^20^ Integrating artificial intelligence (AI) technology to explore the relationship between CEUS's key imaging features and metabolic signatures or immunotherapy effectiveness may enable AI‐based prediction of the efficacy and prognosis of immunotherapy for bladder cancer based on CEUS imaging. In addition, AI technology holds the potential to establish predictive models using big data to overcome diagnostic inconsistencies among radiologists of varying expertise levels, thereby providing greater stability in diagnostic models. Therefore, future research could focus on developing predictive models that utilize AI technology to integrate multiomics data from CEUS and MRI for precise diagnosis of bladder cancer, potentially even predicting the efficacy of bladder cancer immunotherapy.

Finally, since the present study was based on a single center, the sample size needs to be expanded in the future and the effectiveness of combined diagnosis should be validated in multicenter studies. To enhance CEUS's diagnostic accuracy and generalizability in assessing muscle invasion in bladder cancer, future research should incorporate larger multicenter studies involving various radiologists to confirm the present study findings.

In conclusion, the present study demonstrated the clinical value of CEUS in the evaluation of muscle invasion status in bladder cancer patients prior to TURBT or radical cystectomy, which has the potential to aid in clinical decision‐making and improve intervention outcomes in clinical practice.

## 4 MATERIALS AND METHODS

### 4.1 Study design and participants

This prospective study was conducted between April 2020 and September 2021, recruiting patients from Sun Yat‐sen Memorial Hospital, Sun Yat‐sen University (Guangzhou, China). The study adhered to both the principles outlined in the Declaration of Helsinki and the guidelines of the Standards for Reporting of Diagnostic Accuracy (STARD) checklist for the accurate reporting of diagnostic accuracy studies.^21^ The study was approved by the Ethics Committee of Sun Yat‐sen Memorial Hospital, Sun Yat‐sen University (SYSEC‐KY‐KS‐2021‐321), and written informed consent was obtained from all participants. The study is registered with ClinicalTrials.gov (No. NCT05204108).

The inclusion criteria were as follows: (a) age of ≥18 years; (b) histologically or cytologically confirmed primary bladder cancer diagnosis. The exclusion criteria were as follows: (a) participants who were confirmed to have no carcinoma of the bladder by postoperative pathology; (b) patients who underwent therapy, bladder surgery, or received chemotherapy, radiotherapy or immunotherapy treatment; (c) participants diagnosed with tumor recurrence or metastasis; (d) individuals with thickening of the bladder wall due to obstruction and trabeculated bladder (even with diverticula, such as benign prostatic hyperplasia); and (e) those who were allergic to ultrasound contrast agents or could not tolerate CEUS examination, such as patients with recent myocardial infarction, angina pectoris, cardiac insufficiency, severe cardiac arrhythmia, right/left cardiac shunt, severe pulmonary hypertension, uncontrolled systemic hypertension, acute respiratory distress syndrome, or chronic obstructive pulmonary disease. The primary outcome was to evaluate the diagnostic performance of CEUS to determine muscle invasion status in bladder cancer and compare it with surgery‐based pathological results. All bladder cancer patients received CEUS followed by standard TURBT or radical cystectomy regardless of whether they have undergone a previous cystoscopy procedure. Treatment decisions depended primarily on computed tomography/MRI and cystoscopy results, although CEUS outcomes may also have been considered.

### 4.2 CEUS protocol and image analysis

CEUS examinations were performed using four machines at the time of the examination (Aplio i800, Canon Medical Systems Co., Ltd; ACUSON Sequoia, Siemens Medical Solutions; ALOKA ARIETTA 850, Hitachi; Resona 7S, Mindray Bio‐Medical Electronics Co., Ltd.). The i8CX1 (Aplio i800), 5C1 (ACUSON Sequoia), C1‐6 (ALOKA ARIETTA 850), and SC5‐1U (Resona 7S) probes were utilized. CEUS images were captured and analyzed by two experienced radiologists to determine the degree of tumor infiltration. Specifically, the initial CEUS examinations and image analysis were first performed by a radiologist with more than 5 years of experience and were then reassessed under the guidance of a senior radiologist with more than 30 years of experience by reviewing the video. Both radiologists were blinded to the patients’ clinical and histologic information and to one another's results. The mechanical index was maintained between 0.06 and 0.09.

The patients were asked to drink an appropriate amount of water to fill their bladder (optimal volume: 250 ± 50 mL) before the CEUS examination. Poor bladder filling can affect tumor appearance, while overfilling can easily lead to the missed diagnosis of small lesions and interfere with muscular infiltration valuation. A CEUS SonoVue dose of 2.4 mL was used for most patients. All doses were administered as intravenous bolus injections, followed by rinsing with 10.0 mL of normal saline. Most patients needed only one injection. Up to three injections could be given if there were multiple lesions or if additional imaging was required. The observations and stored videos were a minimum of 3 min each. The results were analyzed on a per‐patient basis. Patients enrolled in the analysis with a single or multiple lesions were classified as follows: (a) they were positive if they had at least one lesion showing muscle infiltration as detected with CEUS, and (b) they were negative if they had only one lesion without muscle invasion, or if they had multiple lesions but no muscle invasion as detected with CEUS. In CEUS examinations, the bladder wall muscle was scrutinized for any interruptions to assess whether the tumor had invaded the muscular layer. None of the patients developed an allergy, had their tests interrupted, or died during the trial.

### 4.3 MR protocol and image analysis

MR images, including the individual T2W, DWI, and DCE MRI categories, were analyzed and scored by two experienced radiologists according to the VI‐RADS protocol to determine the degree of tumor infiltration. Both radiologists, each with over 10 years of experience, were blinded to the patients’ clinical and histologic information and one another's results. They independently scored the images and then compared their results. If there were discrepancies between their scores, they reached a consensus through discussion.

### 4.4 Histological analyses

All tissue specimens were fixed in 10% neutral‐buffered formalin, embedded in paraffin, and stained with hematoxylin‐eosin. Specimens were examined by two expert uropathologists to assess the grade and stage of the tumors. Malignant tumors were classified and graded according to the World Health Organization classification.^22^ Tumor stages were defined according to the American Joint Committee on Cancer/Union for International Cancer Control TNM system.^23^ All histopathologic measurements were performed by a pathologist with more than 5 years of experience who was blinded to the participants’ clinical and CEUS data.

### 4.5 Statistical analysis

Differences between categorical variables from different groups were statistically assessed using the χ2 test. If any expected frequency was less than 5, the fisher's exact test was applied. Differences between continuous variables between two groups were statistically assessed by the independent samples *t*‐test or Wilcoxon rank sum test. The results were compared with those of postoperative pathological staging, and the coincidence rate, sensitivity, specificity, and positive and NPVs of CEUS were calculated. The predictive accuracy of CEUS signatures was assessed using receiver operating characteristic analysis by R package pROC. AUC was used to evaluate sensitivity and specificity. DCA was utilized to evaluate the predictive values of CEUS by R package rmda. DCA is a statistical method that assesses the net benefit of a model by comparing it with other available options, allowing to determine the model's clinical usefulness. The DCA results were presented as a curve that plots the net benefit of using a model against the threshold probability of the outcome.^24^ Accuracy, sensitivity, specificity, PPVs, and NPVs were calculated using the caret and epiR packages in R. For all analyses, two‐sided *p* values < 0.05 were considered statistically significant. Statistical analyses were performed using R software (version 4.3.3).

## AUTHOR CONTRIBUTIONS

*Conceptualization, data curation, formal analysis, funding acquisition, methodology, resources, software, validation, visualization, writing—original draft, and writing—review and editing*: Qiyun Ou. *Conceptualization, data curation, project administration, funding acquisition, resources, validation, visualization, writing—original draft, and writing—review and editing*: Weibin Xie. *Conceptualization, formal analysis, funding acquisition, methodology, software, visualization, writing—original draft, and writing—review and editing*: Yunfang Yu. *Resources, validation, and project administration*: Bing Ou. *Data curation and investigation*: Man Luo. *Methodology, software, and visualization*: Yongjian Chen. *Data curation and investigation*: Weiwei Pan. *Resources and validation*: Yiming Lai. *Resources*: Zhuohang Li. *Investigation*: Jianqiu Kong. *Validation*: Zhuo Wu. *Validation*: Jingliang Ruan. *Resources*: Jingjing Han. *Conceptualization, funding acquisition, project administration, resources, and writing—review and editing*: Tianxin Lin. *Conceptualization, funding acquisition, project administration, resources, and writing—review and editing*: Baoming Luo. All authors have read and approved the final manuscript.

## CONFLICT OF INTEREST STATEMENT

All authors declare no conflicts of interest.

## ETHICS STATEMENT

The study was approved by the Ethics Committee of Sun Yat‐sen Memorial Hospital, Sun Yat‐sen University (SYSEC‐KY‐KS‐2021‐321), and written informed consent was obtained from all participants in the study. The study is registered with ClinicalTrials.gov (NO. NCT05204108).

## Supporting information



Supporting Information

Supporting Information

Supporting Information

## Data Availability

The datasets generated or analyzed during the study are not publicly available but are available from the corresponding author on reasonable request.
