# Supplementary material for: Contrast‐enhanced ultrasound enables precision diagnosis of preoperative muscle invasion in bladder cancer: a prospective study
Source: MedComm (2020). 2025 Feb 17;6(3):e70106. doi: 10.1002/mco2.70106 (PMC11832433; doi:10.1002/mco2.70106)
Supplement: Supplementary file 1 — Supporting Information [file MCO2-6-e70106-s001.docx]

**Contrast-Enhanced Ultrasound Enables Precision Diagnosis of Preoperative**

**Muscle Invasion in Bladder Cancer: A Prospective Study**

**Running head:** CEUS Detects Muscle Invasion in Bladder Cancer

Qiyun Ou,^1,2*^ Weibin Xie,^1,3*^ Yunfang Yu,^1,4,5*^ Bing Ou,^1^ Man Luo,^1^ Yongjian Chen,^6^ Weiwei Pan,^1^ Yiming Lai,^1,3^ Zhuohang Li,^1,3^ Jianqiu Kong,^1,3^ Zhuo Wu,^1^ Jingliang Ruan,^1^ Jingjing Han,^1^ Tianxin Lin,^1,3†^ Baoming Luo^1†^

^1^Guangdong Provincial Key Laboratory of Malignant Tumor Epigenetics and Gene Regulation, Department of Ultrasound in Medicine, Department of Urology, Department of Medical Oncology, Sun Yat-sen Memorial Hospital, Sun Yat-sen University, Guangzhou, Guangdong, China

^2^Department of Oncology, Nanfang Hospital, Southern Medical University, Guangzhou, Guangdong, China

^3^Guangdong Provincial Clinical Research Center for Urological Diseases, Guangzhou, Guangdong, China

^4^Shenshan Medical Center, Sun Yat-sen Memorial Hospital, Sun Yat-sen University, Shanwei, Guangdong, China

^5^Faculty of Medicine, Macau University of Science and Technology, Taipa, Macao, China

^6^Department of Medical Oncology, The Third Affiliated Hospital of Sun Yat-sen University, Guangzhou, Guangdong, China

^*^These authors contributed equally and should be considered co-first authors.

^†^These authors contributed equally and should be considered co-corresponding authors.

**Supplement**

**Figure S1. Performance of CEUS in tumor size evaluation for detection of bladder cancer muscle invasion status.** **CEUS, contrast-enhanced ultrasound.**

**
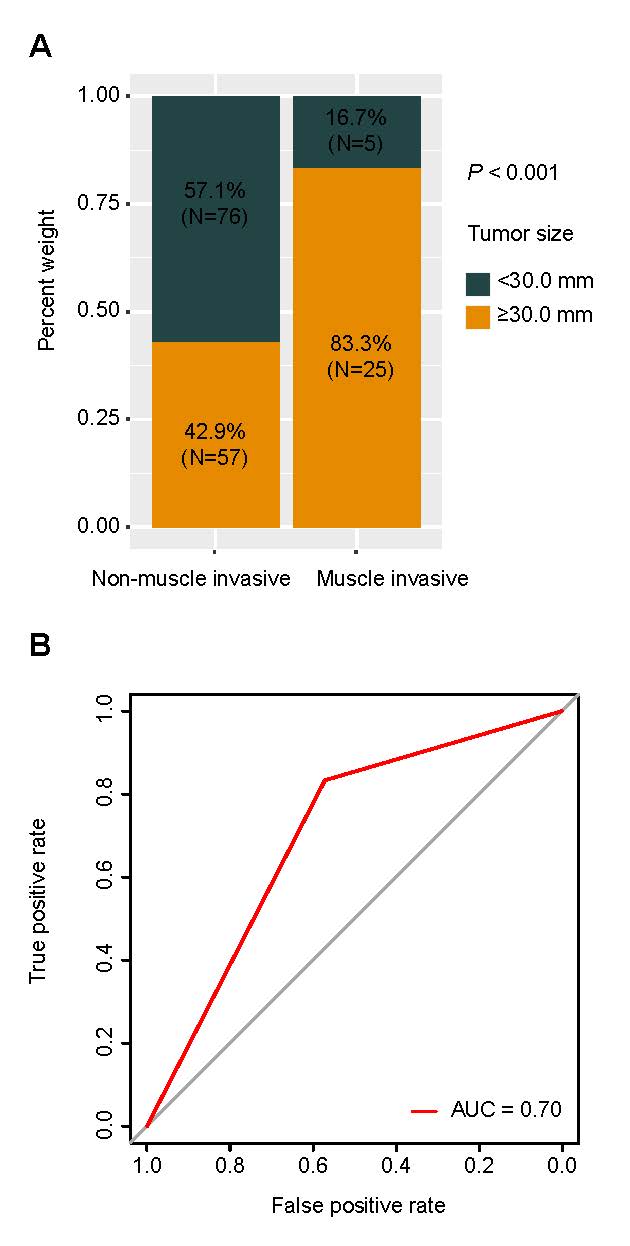
**

(A) The proportions of CEUS in patients with non-muscle invasive versus muscle invasive bladder cancer; (B) CEUS for bladder cancer muscle invasion status estimation; (C) decision curve analysis. CEUS, contrast-enhanced ultrasound.

**Figure S2. Performance of CEUS in tumor base structure status evaluation for detection of bladder cancer muscle invasion status. CEUS, contrast-enhanced ultrasound.**

**
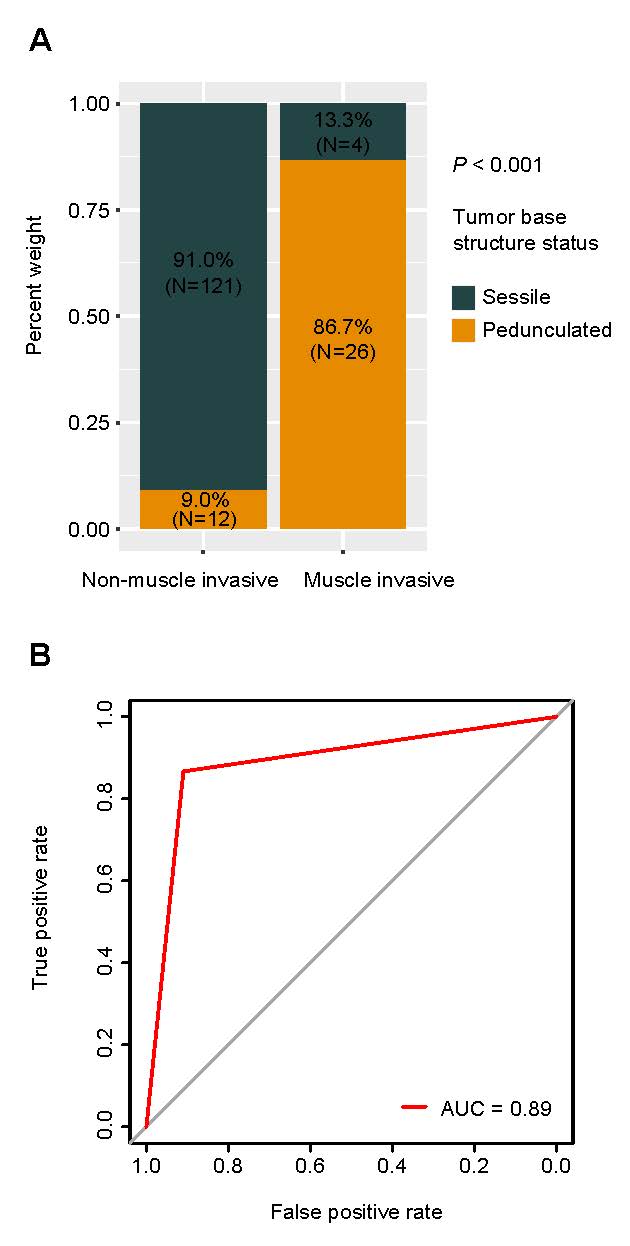
**

(A) The proportions of tumor base structure status (pedunculated and sessile) in patients with non-muscle invasive versus those with muscle invasive; (B) tumor base structure status for bladder cancer muscle invasion status estimation. CEUS, contrast-enhanced ultrasound.

**Figure S3. Performance of CEUS in tumor shape evaluation for detection of bladder cancer muscle invasion status. CEUS, contrast-enhanced ultrasound.**

**
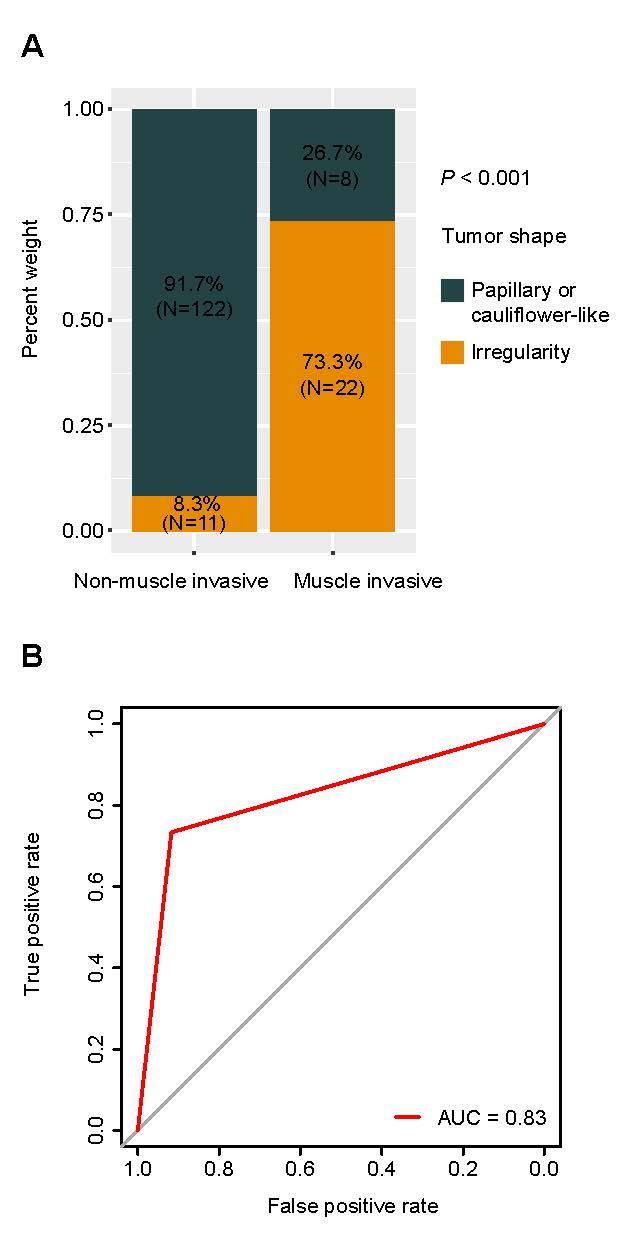
**

(A) The proportions of tumor shape (irregularity and papillary or cauliflower-like) in patients with non-muscle invasive versus those with muscle invasive; (B) tumor shape for bladder cancer muscle invasion status estimation. CEUS, contrast-enhanced ultrasound.

**Figure S4. Performance of CEUS in bladder base continuity status evaluation for detection of bladder cancer muscle invasion status. CEUS, contrast-enhanced ultrasound.**

**
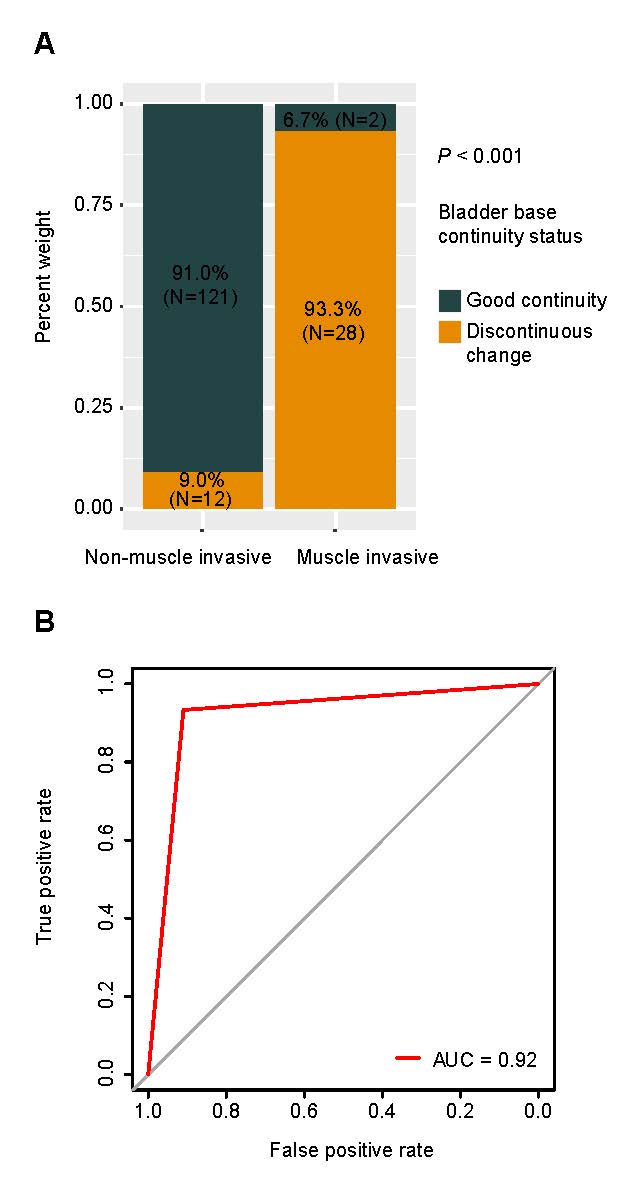
**

(A) The proportions of bladder base continuity status (discontinuous change and good continuity) in patients with non-muscle invasive versus those with muscle invasive; (B) bladder base continuity status for bladder cancer muscle invasion status estimation. CEUS, contrast-enhanced ultrasound.

**Figure S5. Performance of CEUS in tumor base width evaluation for detection of bladder cancer muscle invasion status. CEUS, contrast-enhanced ultrasound.**

**
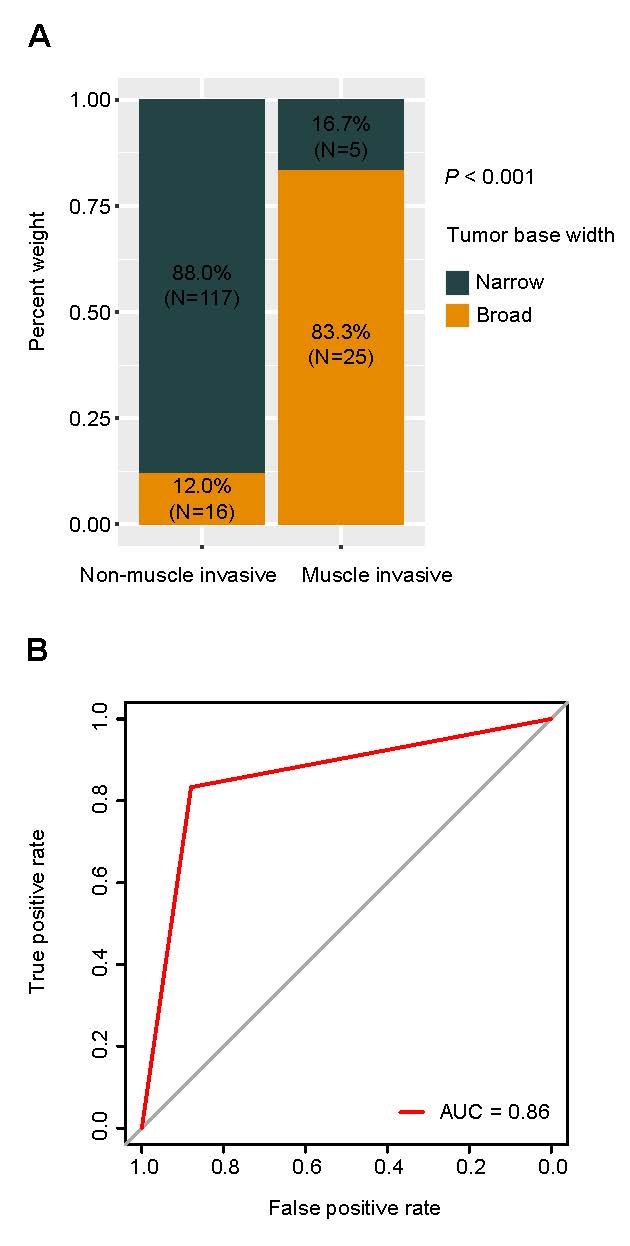
**

(A) The proportions of tumor base width (broad and narrow) in patients with non-muscle invasive versus those with muscle invasive; (B) tumor base width for bladder cancer muscle invasion status estimation. CEUS, contrast-enhanced ultrasound.

**Video legends**

**Video 1.** **CEUS characteristics of non-muscle invasive bladder cancer patients. CEUS, contrast-enhanced ultrasound.**

**Video 2. CEUS characteristics of muscle invasive bladder cancer patients. CEUS, contrast-enhanced ultrasound.**
